# Comparison of the predictive accuracy of multiple definitions of cognitive impairment for incident dementia: a 20-year follow-up of the Whitehall II cohort study

**DOI:** 10.1016/S2666-7568(21)00117-3

**Published:** 2021-07

**Authors:** Marcos D Machado-Fragua, Aline Dugravot, Julien Dumurgier, Mika Kivimaki, Andrew Sommerlad, Benjamin Landré, Aurore Fayosse, Séverine Sabia, Archana Singh-Manoux

**Affiliations:** a Université de Paris, Inserm U1153, Epidemiology of Ageing and Neurodegenerative Diseases, Paris, France; b Department of Epidemiology and Public Health, University College London, London, UK; c Division of Psychiatry, University College London, London, UK; d Cognitive Neurology Center, Saint Louis-Lariboisiere-Fernand Widal Hospital, AP-HP, Université de Paris, Paris, France; e Helsinki Institute of Life Sciences, University of Helsinki, Helsinki, Finland; f Camden and Islington NHS Foundation Trust, London, UK

## Abstract

**Background:**

Studies generally use cognitive assessment done at one timepoint to define cognitive impairment in order to examine conversion to dementia. Our objective was to examine the predictive accuracy and conversion rate of seven alternate definitions of cognitive impairment for dementia.

**Methods:**

In this prospective study, we included participants from the Whitehall II cohort study who were assessed for cognitive impairment in 2007–09 and were followed up for clinically diagnosed dementia. Algorithms based on poor cognitive performance (defined using age-specific and sex-specific thresholds, and subsequently thresholds by education or occupation levels) and objective cognitive decline (using data from cognitive assessments in 1997–99, 2002–04, and 2007–09) were used to generate seven alternate definitions of cognitive impairment. We compared predictive accuracy using Royston's *R*^2^, the Akaike information criterion (AIC), sensitivity, specificity, and Harrell's C-statistic.

**Findings:**

5687 participants, with a mean age of 65·7 years (SD 5·9) in 2007–09, were included and followed up for a median of 10·5 years (IQR 10·1–10·9). Over follow-up, 270 (4·7%) participants were clinically diagnosed with dementia. Cognitive impairment defined using both cognitive performance and decline had higher hazard ratios (from 5·08 [95% CI 3·82–6·76] to 5·48 [4·13–7·26]) for dementia than did definitions based on cognitive performance alone (from 3·25 [2·52–4·17] to 3·39 [2·64–4·36]) and cognitive decline alone (3·01 [2·37–3·82]). However, all definitions had poor predictive performance (C-statistic ranged from 0·591 [0·565–0·616] to 0·631 [0·601–0·660]), primarily due to low sensitivity (21·6–48·4%). A predictive model containing age, sex, and education without measures of cognitive impairment had better predictive performance (C-statistic 0·783 [0·758–0·809], sensitivity 74·2%, specificity 72·2%) than all seven definitions of cognitive impairment (all p<0·0001).

**Interpretation:**

These findings suggest that cognitive impairment in early old age might not be useful for dementia prediction, even when it is defined using longitudinal data on cognitive decline and thresholds of poor cognitive performance additionally defined by education or occupation.

**Funding:**

National Institutes of Health, UK Medical Research Council.

## Introduction

Late-onset dementias are complex diseases with a long preclinical phase, with pathophysiological hallmarks appearing 15–20 years before clinical symptoms.^1^ It has been estimated that even small delays in onset could substantially reduce the burden of Alzheimer's disease and related dementias and could result in substantial savings to health-care systems.^2^ There are no established methods of early identification of individuals at risk of dementia. Alongside biological models of Alzheimer's disease, there is considerable research on understanding changes in clinical trajectory, primarily cognitive function, leading to dementia. The intermediate stage between healthy ageing and dementia has been studied using concepts such as mild cognitive impairment (MCI)^3^ to identify the intermediate, transitional phase between normal cognitive ageing and dementia onset.

The criteria for these diagnostic constructs of intermediate stages typically include subjective cognitive or memory complaints reported by the patient or an informant or objective loss of cognition from a previous state, measurable poor cognitive performance, and absence of a dementia diagnosis.^3^, ^4^, ^5^ The optimal definition of loss of cognition from a previous state remains unclear; most studies rely on clinical judgment but repeated cognitive testing is thought to be more informative.^6^, ^7^ Most studies on cognitive impairment show that impairment is associated with an increased risk of dementia.^3^, ^4^, ^8^, ^9^ Much research on cognitive impairment has focused on conversion rates, reflecting the percentage of people with cognitive impairment who progress to dementia, rather than on assessments of the predictive accuracy of cognitive impairment for dementia, which might be more meaningful for assessment of the risk of dementia and more informative in clinical settings. In addition, most studies rely on poor cognitive performance at a single timepoint rather than cognitive decline in the assessment of cognitive impairment.

**Research in context**

**Evidence before this study**

We did a comprehensive search of studies on progression from cognitive impairment to dementia using PubMed, including all studies from database inception until Jan 20, 2021. We used the following search terms: “dementia”, “cognitive impairment”, “cognitive decline”, “poor cognitive performance”, “progression”, and “prediction”. Our search showed that most studies used a single cognitive assessment to ascertain cognitive impairment and then examined rate of conversion to dementia, often using a short follow-up. While this method suggests associations between cognitive impairment and dementia, it does not allow conclusions to be drawn on the validity of cognitive impairment in predicting dementia.

**Added value of this study**

To our knowledge, this is the first study to use prediction statistics in addition to conversion rates to compare the validity of seven alternate definitions of cognitive impairment for dementia prediction. The results show that demographic variables (age, sex, and education) on their own predicted dementia better than cognitive impairment, irrespective of inclusion of cognitive decline along with poor cognitive performance in the definition of cognitive impairment. These findings suggest that cognitive impairment might be necessary but not sufficient to predict dementia.

**Implications of all the available evidence**

The ageing of populations and the corresponding increase in dementia burden makes it urgent to identify individuals who could benefit from early interventions to delay or prevent dementia. Cognitive impairment is widely used to identify such individuals. Our results show cognitive impairment has poor predictive accuracy for dementia, implying that further research is required to identify individuals at risk of dementia.

We therefore tested the hypothesis that inclusion of objective measures of cognitive decline, using serial assessments of cognitive function, in addition to a measure of poor cognitive performance at one timepoint improves predictive accuracy of cognitive impairment for dementia. Poor cognitive performance was defined using age-specific and sex-specific thresholds, and then using educational attainment and occupational position at age 50 years because age, sex, and socioeconomic factors are known to shape cognitive trajectories.^10^, ^11^ The predictive accuracy of seven different cognitive impairment definitions was examined for the primary outcome of dementia and the secondary outcome of mortality.

## Methods

### Study population

Data are from the ongoing Whitehall II cohort study, which was established in 1985 among 10 308 British civil servants (6895 men and 3413 women) aged 35–55 years.^12^ A cognitive test battery was introduced to the study in 1997–99 and repeated in 2002–04, 2007–09, 2012–13, and 2015–16. In addition to data collection within the study, data over the follow-up were available using linkage to electronic health records of the UK National Health Service (NHS) for all but ten of the 10 308 participants recruited to the study. There were no exclusion criteria. Participants gave informed written consent at each contact and the latest ethical approval was from the NHS London Harrow Research Ethics Committee (reference number 85/0938).

### Measures

The cognitive test battery was composed of tests of memory (assessed using a 20-word free recall test done in 2 min), language (using a measure of phonemic, in which participants were asked to produce as many words as they could starting with “s” in 1 min, and a measure of semantic fluency, in which participants named as many animals as they could in 1 min), executive function (using the 10-min Alice Heim 4-I test,^13^ which includes a set of 65 verbal and mathematical reasoning items of increasing difficulty), and the Mini Mental State Examination (MMSE).^14^

Poor cognitive performance in 2007–09 was defined as an MMSE score of less than 24,^15^ or scores on other cognitive tests (memory, language, or executive function) 1·5 SD below the mean,^16^ calculated from the distribution of each cognitive test score within the study population using the following steps (syntax provided in appendix p 2): (1) standardise measures of memory, language, and executive function; (2) regress each cognitive test score for every individual using age (at date of clinical assessment in 2007–09) and sex; (3) extract residuals (observed – predicted test score); (4) calculate the root-mean-squared error; (5) standardise the residuals using the root-mean-squared error; and (6) apply the 1·5-SD threshold to the standardised residuals.

The procedure described above was repeated by adding education (three-level variable categorised as university or higher degree, higher secondary school [ie, A levels], or lower secondary school [ie, O levels] or less) and then occupation (three-level variable categorised as high, intermediate, or low, using the British Civil Service grade of employment at age 50 years) to the regression model to establish the various thresholds for poor cognitive performance.

Cognitive decline was defined using slopes of change in cognitive test scores with data from 1997–99, 2002–04, and 2007–09, in participants with two or more waves of data. Cognitive decline was defined as being in the lowest tenth percentile of the slope of change for at least one cognitive domain or below the 20th percentile for more than one domain.^16^

Seven definitions of cognitive impairment were determined using the following criteria: (1) age-specific and sex-specific threshold for poor cognitive performance; (2) cognitive decline; (3) age-specific and sex-specific threshold for poor cognitive performance plus cognitive decline; (4) age-specific, sex-specific, and education-specific threshold for poor cognitive performance; (5) age-specific, sex-specific, and education-specific threshold for poor cognitive performance plus cognitive decline; (6) age-specific, sex-specific, and occupation-specific threshold for poor cognitive performance; and (7) age-specific, sex-specific, and occupation-specific threshold for poor cognitive performance plus cognitive decline.

### Outcomes

For our primary outcome of dementia, dementia ascertainment was undertaken using linkage to Hospital Episode Statistics (HES), the Mental Health Services Data Set, and the British national mortality register using the International Classification of Diseases, Tenth Revision codes F00–F03, F05·1, G30, and G31. Record linkage for dementia was available until March 31, 2019. The HES contains clinical diagnoses from inpatient, outpatient, and emergency departments and has a sensitivity of 78·0% and specificity of 92·0%.^17^ The Mental Health Services Data Set contains dementia diagnoses from inpatient, outpatient, and community mental health services, including memory services. The British national mortality register collects information about cause-specific mortality. Date of dementia was set at the first record of dementia diagnosis in any of these three databases.

For our secondary outcome of mortality, the personal NHS identification number was used to identify participants who died during follow-up. Mortality data were collected until Sept 31, 2019, from the British national mortality register.

### Statistical analysis

Participants' characteristics in 2007–09 were examined as a function of concurrent cognitive impairment status as well as dementia status at the end of follow-up. Differences in sociodemographic variables and cognitive impairment status were assessed using the χ^2^ test and Student's *t* test, as appropriate. Cohen's κ coefficients were used to describe the agreement between the seven definitions of cognitive impairment.

We calculated the dementia rate per 1000 person-years for each cognitive impairment definition. Cox proportional hazard regression analysis was used to estimate the hazard ratio (HR) for the association between cognitive impairment and incidence of dementia for all seven definitions. The start of follow-up was the date of each participant's 2007–09 clinical examination and participants were censored at date of record of dementia, death, or March 31, 2019, whichever came first. We accounted for competing risk of death using cause-specific hazard models by censoring at date of death for participants who died during follow-up.^18^ The predictive accuracy of the seven cognitive impairment definitions were assessed using Royston's modified *R*^2^ for survival data,^19^ the Akaike information criterion (AIC), sensitivity and specificity using survival models, and Harrell's C-statistic for survival models. *R*^2^, with 95% CIs calculated using 2000 bootstrap replications, was used to measure the overall performance, with higher values indicating greater explained variation. AIC is a measure of the relative goodness of fit of a statistical model, where lower values indicate better model fit. Differences in the AIC of 10 or more between definitions were considered to be meaningful. Sensitivity and specificity were included as measures of classification accuracy. Harrell's C-statistic was calculated with 95% CIs to evaluate discrimination,^20^ and was compared between the seven definitions using a non-parametric approach with definition 1 (age and sex threshold for poor cognitive performance) as the reference. In these analyses, we also compared Harrell's C-statistic obtained for the seven cognitive impairment definitions with that obtained using only age, sex, and education to predict dementia.

The analyses described above were repeated using mortality as the outcome. The start of follow-up in these analyses was also the date of each participant's 2007–09 clinical examination, and participants were censored at date of death or Sept 31, 2019, whichever came first.

We did six sensitivity analyses to assess the robustness of our results. First, we used a three-category variable (no cognitive impairment, only poor cognitive performance, or poor cognitive performance and cognitive decline) to assess the importance of cognitive decline in cognitive impairment when both poor cognitive performance and cognitive decline are included in the definition (definitions 3, 5, and 7), using age, sex, and then education and occupation thresholds as described in the main analyses. Second, to address the effect of selection biases due to missing data, we repeated the main analysis using inverse probability weighting to reflect the study population at the start of the cohort study (ie, the year 1985). Of 9362 participants who were alive in 2007–09 (start of follow-up in these analyses) and did not have a dementia diagnosis, we first calculated the probability of being included in the present study using data from the start of the cohort study on sociodemographic factors, health behaviours, cardiometabolic risk factors, and mental health and data on chronic conditions over the follow-up period (1985–2019). The inverse of these probabilities was used to weight the analyses of the association between cognitive impairment and dementia. Third, we changed the thresholds used to define poor cognitive performance (using 1 SD and 1·96 SD instead of 1·5 SD) to assess whether the observed association between cognitive impairment and dementia varied as a function of the threshold used to define cognitive impairment. Fourth, we restricted analyses to participants aged 65 years or older at their 2007–09 cognitive impairment assessment to examine whether the predictive accuracy of cognitive impairment for incident dementia was different in the older age group. Fifth, to account for reversion from cognitive impairment to normal cognitive status between 1997 and 2009, we used a three-category variable (no cognitive impairment, no cognitive impairment but previous poor cognitive performance, or current cognitive impairment) to assess the association between reversion and the risk of dementia. Reversion was defined as cases in which a participant was classified as having no cognitive impairment after being identified as cognitively impaired in any previous examination. Finally, we added established risk factors for dementia^21^—namely, age, sex, and education—to the prediction model to assess improvement in the predictive accuracy of cognitive impairment definitions for dementia. The optimal cutoff point for sensitivity and specificity was established by maximising the Youden index (calculated as sensitivity + specificity – 1).^22^

All analyses were done with Stata version 15.0. Two-sided p<0·05 was considered statistically significant in all analyses.

### Role of the funding source

The funders had no role in the study design, data collection, data analysis, data interpretation, or writing of the report.

## Results

Of the 10 308 participants in the Whitehall II cohort study, 954 (9·3%) died and 2593 (25·2%) were lost to follow-up between 1985 and 2007, before the assessment of cognitive impairment (2007–09). We also excluded 1064 (10·3%) participants with missing cognitive data and ten (0·1%) prevalent cases of dementia at baseline (2007–09), leading to 5687 participants included in the analysis (figure). The average age of participants at cognitive impairment assessment was 65·7 years (SD 5·9), and 1552 (27·3%) were women (table 1). Compared with all participants, those who were identified as having cognitive impairment were generally older, had lower education and occupational attainment, and had lower cognitive test scores and greater 10-year decline in all cognitive domains.

**Figure:**
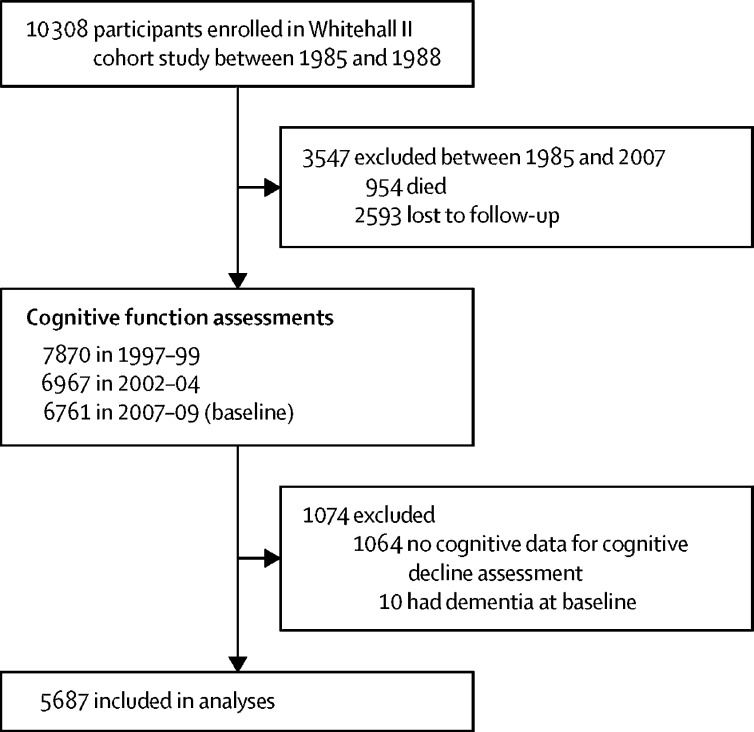
Flow chart of sample selection

**Table 1:** Characteristics of participants by cognitive impairment definition

		**All participants (n=5687)**	**Participants with cognitive impairment**						
			Definition 1 (n=821)	Definition 2 (n=1485)	Definition 3 (n=338)	Definition 4 (n=837)	Definition 5 (n=347)	Definition 6 (n=826)	Definition 7 (n=354)
**Demographics**									
Age, years		65·7 (5·9)	65·8 (5·9)	67·0 (6·0)	67·2 (6·0)	66·0 (6·0)	67·2 (6·1)	65·8 (6·1)	67·2 (6·1)
Sex									
	Female	1552 (27·3%)	264 (32·2%)	454 (30·6%)	113 (33·4%)	253 (30·2%)	108 (31·1%)	241 (29·2%)	109 (30·8%)
	Male	4135 (72·7%)	557 (67·8%)	1031 (69·4%)	225 (66·6%)	584 (69·8%)	239 (68·9%)	585 (70·8%)	245 (69·2%)
Education									
	Lower secondary school or less	2374 (41·7%)	487 (59·3%)	649 (43·7%)	191 (56·5%)	347 (41·5%)	150 (43·2%)	400 (48·4%)	171 (48·3%)
	Higher secondary school	1556 (27·4%)	190 (23·1%)	394 (26·5%)	85 (25·2%)	222 (26·5%)	96 (27·7%)	205 (24·8%)	86 (24·3%)
	University degree or higher	1757 (30·9%)	144 (17·6%)	442 (29·8%)	62 (18·3%)	268 (32·0%)	101 (29·1%)	221 (26·8%)	97 (27·4%)
Occupation									
	Low	609 (10·7%)	248 (30·2%)	190 (12·8%)	98 (29·0%)	226 (27·0%)	86 (24·8%)	119 (14·4%)	56 (15·8%)
	Intermediate	2530 (44·5%)	425 (51·8%)	619 (41·7%)	154 (45·6%)	401 (47·9%)	151 (43·5%)	356 (43·1%)	141 (39·8%)
	High	2548 (44·8)	148 (18·0%)	676 (45·5%)	86 (25·4%)	210 (25·1%)	110 (31·7%)	351 (42·5%)	157 (44·4%)
**Cognitive scores**									
Scores at baseline (2007–09)									
	Memory (range 0–20)	6·21 (2·21)	4·32 (2·21)	5·51 (2·19)	3·86 (2·22)	4·38 (2·29)	3·98 (2·35)	4·48 (2·25)	4·05 (2·30)
	Language (range 0–35)	15·34 (3·46)	11·64 (3·44)	14·56 (3·63)	11·56 (3·97)	11·78 (3·48)	11·63 (3·96)	12·03 (3·51)	11·83 (3·85)
	Executive function (range 0–65)	43·92 (10·82)	30·38 (11·34)	41·31 (11·27)	31·05 (11·78)	31·35 (11·74)	32·14 (12·04)	33·06 (11·84)	33·30 (11·86)
	Proportion with MMSE <24 (n=45)	0·8%	5·5%	3·0%	13·3%	5·4%	13·0%	5·5%	12·7%
10-year change									
	Memory	−0·32 (1·15)	−0·56 (1·39)	−1·05 (1·46)	−1·23 (1·58)	−0·56 (1·39)	−1·22 (1·59)	−0·62 (1·35)	−1·27 (1·50)
	Language	−0·37 (0·79)	−0·49 (1·01)	−0·87 (1·02)	−0·92 (1·27)	−0·51 (1·01)	−0·94 (1·26)	−0·54 (1·02)	−0·97 (1·25)
	Executive function	−0·27 (0·60)	−0·45 (0·69)	−0·60 (0·79)	−0·78 (0·84)	−0·45 (0·69)	−0·75 (0·85)	−0·48 (0·70)	−0·79 (0·85)

Data are mean (SD), n (%), or %. The definitions of cognitive impairment are as follows: (1) age and sex threshold for poor cognitive performance; (2) cognitive decline; (3) age and sex threshold for poor cognitive performance plus cognitive decline; (4) age, sex, and education threshold for poor cognitive performance; (5) age, sex, and education threshold for poor cognitive performance plus cognitive decline; (6) age, sex, and occupation threshold for poor cognitive performance; and (7) age, sex, and occupation threshold for poor cognitive performance plus cognitive decline. MMSE=Mini Mental State Examination.

Over a median follow-up of 10·5 years (IQR 10·1–10·9), 270 (4·7%) participants were diagnosed with dementia and 649 (11·4%) died. At baseline clinical assessments (2007–09), participants who were subsequently diagnosed with dementia were older, had lower educational level and occupational attainment, and had higher prevalence of cognitive impairment (table 2). In addition, the cumulative conversion ranged from 9% to 18% for the seven definitions of cognitive impairment.

**Table 2:** Sample characteristics in 2007–09 by dementia status at the end of follow-up (March 31, 2019)

		**No dementia (n=5417)**	Dementia*(n=270)	**p value**†
Percentage of cohort		95·2%	4·8%	..
Age at baseline, years		65·5 (5·8)	71·4 (4·9)	<0·0001
Sex				0·032
	Female	1463 (27·0%)	89 (33·0%)	..
	Male	3954 (73·0%)	181 (67·0 %)	..
Education				<0·0001
	Lower secondary school or less	2224 (41·1%)	150 (55·6%)	..
	Higher secondary school	1497 (27·6%)	59 (21·9%)	..
	University degree or higher	1696 (31·3%)	61 (22·6%)	..
Occupation				<0·0001
	Low	552 (10·2%)	57 (21·1%)	..
	Intermediate	2414 (44·6%)	116 (43·0%)	..
	High	2451 (45·2%)	97 (35·9%)	..
Cognitive impairment definitions				
	(1) Age and sex threshold for poor cognitive performance	730 (13·5%)	91 (33·7%)	<0·0001
	(2) Cognitive decline	1351 (24·9%)	134 (49·6%)	<0·0001
	(3) Age and sex threshold for poor cognitive performance plus cognitive decline	278 (5·1%)	60 (22·2%)	<0·0001
	(4) Age, sex, and education threshold for poor cognitive performance	742 (13·7%)	95 (35·2%)	<0·0001
	(5) Age, sex, and education threshold for poor cognitive performance plus cognitive decline	284 (5·2%)	63 (23·3%)	<0·0001
	(6) Age, sex, and occupation threshold for poor cognitive performance	734 (13·6%)	92 (34·1%)	<0·0001
	(7) Age, sex, and occupation threshold for poor cognitive performance plus cognitive decline	293 (5·4%)	61 (22·6%)	<0·0001

Data are n (%), %, or mean (SD), unless otherwise specified.

* Dementia subtypes were Alzheimer's disease (n=108), vascular dementia (n=43), Parkinson's dementia (n=9), mixed Alzheimer's and vascular dementia (n=14), mixed vascular and Parkinson's dementia (n=2), mixed Alzheimer's and Parkinson's dementia (n=4), and other or missing subtype (n=90).

† p values for differences in χ^2^ test (categorical data) or Student's *t* test (continuous).

The agreement between the seven definitions of cognitive impairment showed that the definition based only on cognitive decline (definition 2) had poor agreement with definitions based only on cognitive performance (definition 1: κ=0·13; definition 4: κ=0·14; and definition 6: κ=0·15; appendix p 3). Definitions based on cognitive performance alone (definitions 1, 4, and 6) had considerable agreement with each other, with κ ranging from 0·69 to 0·79. Similarly, definitions based on both cognitive performance and cognitive decline (definitions 3, 5, and 7) had high agreement, with κ ranging from 0·78 to 0·86.

The rate of dementia per 1000 person-years was higher in participants with cognitive impairment than in those without cognitive impairment (table 3). These differences were larger when cognitive impairment was defined using both cognitive performance and cognitive decline (definitions 3, 5, and 7) than when using only cognitive performance (definitions 1, 4, and 6) or only cognitive decline (definition 2; table 3). This pattern of results was reflected in results from Cox regression, with the highest HR for participants with cognitive impairment for definition 5 (age, sex, and education threshold for poor cognitive performance plus cognitive decline) and lowest for definition 2 (cognitive decline only; table 3).

**Table 3:** Predictive performance of seven cognitive impairment definitions for dementia

		**Cases/participants**	**Dementia rate per 1000 person-years**	**Mean years of follow-up (SD)**	**HR (95% CI)**	**R^2^ (95% CI)**	**AIC**	**Δ AIC**	**Sensitivity**	**Specificity**	**C-statistic (95% CI)**	**p value for difference in C-statistic**	
												Definition 1 (ref)	Demographic model (ref)
**Cognitive impairment definitions**													
Definition 1													
	No cognitive impairment	179/4866	3·7	10·1 (1·7)	1 (ref)	0·155 (0·088–0·238)	4527·9	0 (ref)	32·9%	86·6%	0·608 (0·580–0·637)	NA	<0·0001
	Cognitive impairment	91/821	11·6	9·5 (2·4)	3·26 (2·53–4·19)	..	..	..	..	..	..	..	..
Definition 2													
	No cognitive impairment	136/4202	3·2	10·1 (1·7)	1 (ref)	0·169 (0·102–0·248)	4521·4	−6·5	48·4%	75·1%	0·631 (0·601–0·660)	0·23	<0·0001
	Cognitive impairment	134/1485	9·4	9·6 (2·3)	3·01 (2·37–3·82)	..	..	..	..	..	..	..	..
Definition 3													
	No cognitive impairment	210/5349	3·9	10·1 (1·8)	1 (ref)	0·200 (0·117–0·292)	4506·3	−21·6	21·8%	94·9%	0·592 (0·566–0·617)	0·09	<0·0001
	Cognitive impairment	60/338	19·8	9·0 (2·9)	5·30 (3·98–7·07)	..	..	..	..	..	..	..	..
Definition 4													
	No cognitive impairment	175/4850	3·6	10·1 (1·7)	1 (ref)	0·169 (0·099–0·257)	4521·0	−6·9	34·3%	86·4%	0·612 (0·583–0·641)	0·64	<0·0001
	Cognitive impairment	95/837	11·9	9·5 (2·5)	3·39 (2·64–4·36)	..	..	..	..	..	..	..	..
Definition 5													
	No cognitive impairment	207/5340	3·9	10·1 (1·8)	1 (ref)	0·214 (0·130–0·311)	4499·2	−28·7	22·4%	94·8%	0·595 (0·569–0·621)	0·25	<0·0001
	Cognitive impairment	63/347	20·2	9·0 (2·9)	5·48 (4·13–7·26)	..	..	..	..	..	..	..	..
Definition 6													
	No cognitive impairment	178/4861	3·6	10·1 (1·8)	1 (ref)	0·155 (0·088–0·234)	4527·7	−0·2	32·5%	86·5%	0·608 (0·579–0·637)	0·99	<0·0001
	Cognitive impairment	92/826	11·6	9·6 (2·4)	3·25 (2·52–4·17)	..	..	..	..	..	..	..	..
Definition 7													
	No cognitive impairment	209/5333	3·9	10·1 (1·8)	1 (ref)	0·194 (0·115–0·285)	4509·0	−18·9	21·6%	94·6%	0·591 (0·565–0·616)	0·15	<0·0001
	Cognitive impairment	61/354	19·0	9·0 (2·9)	5·08 (3·82–6·76)	..	..	..	..	..	..	..	..
**Demographic model**													
Age, sex, and education		270/5687	NA	NA	NA	0·543 (0·465–0·626)	4312·6	−215·3	74·2%	72·2%	0·783 (0·758–0·809)	<0·0001	NA

The definitions of cognitive impairment are as follows: (1) age and sex threshold for poor cognitive performance; (2) cognitive decline; (3) age and sex threshold for poor cognitive performance plus cognitive decline; (4) age, sex, and education threshold for poor cognitive performance; (5) age, sex, and education threshold for poor cognitive performance plus cognitive decline; (6) age, sex, and occupation threshold for poor cognitive performance; and (7) age, sex, and occupation threshold for poor cognitive performance plus cognitive decline. HR=hazard ratio. AIC=Akaike information criterion. NA=not applicable.

Definition 5 also had the highest explained variance and the best model fit (table 3). The cognitive impairment definitions that include both poor cognitive performance and cognitive decline had better goodness of fit in terms of *R*^2^ and AIC, with more than a 10-point decline in AIC for definitions 3, 5, and 7, compared with definition 1 (age and sex threshold for cognitive performance; table 3). The C-statistic for definition 1 was modest, and C-statistics for the other definitions of cognitive impairment did not significantly differ from the C-statistic for definition 1 (all p>0·05), suggesting similar discrimination across definitions (table 3). Sensitivity of the definitions ranged from 21·6% to 48·4% and specificity from 75·1% to 94·9%, with definitions that included both cognitive performance and decline (definitions 3, 5, and 7) showing lower sensitivity but greater specificity (table 3). The prediction model including only demographic variables (age, sex, and education) had a significantly higher C-statistic than any of the seven cognitive impairment measures (table 3).

Significant associations with mortality were observed for all seven cognitive impairment definitions (table 4). The *R*^2^ for mortality for all seven definitions were lower than the *R*^2^ observed for dementia (Table 3, Table 4). Cognitive impairment defined using only cognitive decline (definition 2) had the best overall fit (*R*^2^ and AIC) and the best discrimination (C-statistic), significantly better than definition 1 (table 4).

**Table 4:** Predictive performance of seven cognitive impairment definitions for mortality

		**Deaths/participants**	**Death rate per 1000 person-years**	**Mean years of follow-up (SD)**	**HR (95% CI)**	**R^2^ (95% CI)**	**AIC**	**Δ AIC**	**Sensitivity**	**Specificity**	**C-statistic (95% CI)**	**p value for difference in C-statistic**	
												Definition 1 (ref)	Demographic model (ref)
**Cognitive impairment definitions**													
Definition 1													
	No cognitive impairment	508/4866	9·8	10·6 (1·8)	1 (ref)	0·027 (0·011–0·050)	11058·3	0 (ref)	22·1%	86·5%	0·540 (0·524–0·556)	NA	<0·0001
	Cognitive impairment	141/821	16·7	10·3 (2·2)	1·71 (1·42–2·06)	..	..	..	..	..	..	..	..
Definition 2													
	No cognitive impairment	400/4202	8·9	10·7 (1·7)	1 (ref)	0·050 (0·026–0·080)	11 033·2	−25·1	38·4%	75·5%	0·566 (0·547–0·585)	0·024	<0·0001
	Cognitive impairment	249/1485	16·3	10·3 (2·1)	1·84 (1·57–2·16)	..	..	..	..	..	..	..	..
Definition 3													
	No cognitive impairment	572/5349	10·1	10·6 (1·8)	1 (ref)	0·036 (0·015–0·066)	11 048·4	−9·9	12·2%	94·9%	0·533 (0·520–0·545)	0·21	<0·0001
	Cognitive impairment	77/338	22·9	9·9 (2·5)	2·31 (1·82–2·93)	..	..	..	..	..	..	..	..
Definition 4													
	No cognitive impairment	505/4850	9·8	10·6 (1·8)	1 (ref)	0·028 (0·010–0·052)	11 057·4	−0·9	22·7%	86·3%	0·542 (0·526–0·557)	0·79	<·0001
	Cognitive impairment	144/837	16·7	10·3 (2·2)	1·71 (1·43–2·07)	..	..	..	..	..	..	..	..
Definition 5													
	No cognitive impairment	569/5340	10·1	10·6 (1·8)	1 (ref)	0·038 (0·016–0·067)	11 045·8	−12·5	12·7%	94·7%	0·534 (0·522–0·547)	0·36	<0·0001
	Cognitive impairment	80/347	23·2	10·0 (2·5)	2·35 (1·86–2·96)	..	..	..	..	..	..	..	..
Definition 6													
	No cognitive impairment	514/4861	10·0	10·6 (1·8)	1 (ref)	0·020 (0·518–0·549)	11 065·7	7·4	21·0%	86·3%	0·534 (0·518–0·549)	0·29	<0·0001
	Cognitive impairment	135/826	15·8	10·3 (2·2)	1·60 (1·32–1·93)	..	..	..	..	..	..	..	..
Definition 7													
	No cognitive impairment	569/5333	10·1	10·6 (1·8)	1 (ref)	0·037 (0·016–0·065)	11 047·8	−10·5	12·7%	94·6%	0·534 (0·521–0·546)	0·35	<0·0001
	Cognitive impairment	80/354	22·7	10·0 (2·5)	2·29 (1·81–2·90)	..	..	..	..	..	..	..	..
**Demographic model**													
Age, sex, and education		649/5687	NA	NA	NA	0·322 (0·267–0·385)	10 718·0	−340·3	64·8%	71·5%	0·708 (0·688–0·729)	<0·0001	NA

The definitions of cognitive impairment are as follows: (1) age and sex threshold for poor cognitive performance; (2) cognitive decline; (3) age and sex threshold for poor cognitive performance plus cognitive decline; (4) age, sex, and education threshold for poor cognitive performance; (5) age, sex, and education threshold for poor cognitive performance plus cognitive decline; (6) age, sex, and occupation threshold for poor cognitive performance; and (7) age, sex, and occupation threshold for poor cognitive performance plus cognitive decline. HR=hazard ratio. AIC=Akaike information criterion. NA=not applicable.

Sensitivity analyses showed systematically stronger associations with dementia when cognitive decline was included in the definition of cognitive impairment (p<0·0001 for comparison of HRs; appendix p 4). Inverse probability weighting to account for missing data yielded results similar to those reported in the primary analysis (table 3), with the highest HR for definition 5 (4·99, 95% CI 3·69–6·74) and the lowest for definition 2 (2·70, 2·09–3·48; appendix p 5). When thresholds of 1 SD rather than 1·5 SD were used, the highest HR was found for definition 7 (4·17, 3·24–5·37) and lowest for definition 4 (2·63, 2·07–3·34; appendix p 6). For the 1·96-SD threshold, the highest HR was for definition 3 (7·09, 5·07–9·93) and lowest for definition 2 (3·01, 2·37–3·82; appendix p 7). Analyses restricted to the 2837 participants aged 65 years or older at their 2007–09 assessment showed similar results to the main analysis, with definition 5 having the highest HR (4·84, 3·58–6·55) and definition 2 the lowest (2·75, 2·12–3·56; appendix p 8). The risk of dementia in participants with unstable cognitive impairment (reversion from cognitive impairment to no cognitive impairment status) was similar to the risk in participants without cognitive impairment (appendix p 9).

Finally, adding age, sex, and education to cognitive impairment measures considerably improved the predictive accuracy of all definitions (appendix p 10). For example, definition 1 (age and sex threshold for cognitive performance) had its C-statistic change from 0·608 (95% CI 0·580–0·637) to 0·807 (0·783–0·831), with improvement in *R*^2^ from 0·155 (0·088–0·238) to 0·643 (0·565–0·728) and in sensitivity from 32·9% to 77·2% (table 3; appendix p 10). In these analyses, predictive models performed equally well across all definitions of cognitive impairment.

## Discussion

This prospective study has five key findings. First, cognitive impairment defined using an objective measure of 10-year cognitive decline along with poor cognitive performance at one timepoint was more strongly associated with dementia compared with definitions using only poor cognitive performance or only cognitive decline. Second, the discrimination and predictive accuracy of all cognitive impairment definitions for dementia was modest, characterised by high specificity but modest sensitivity. Most studies on cognitive impairment are done in older adults,^23^, ^24^ and restricting our analyses to participants older than 65 years did not alter the main findings. Use of alternate thresholds of poor cognitive performance and cognitive decline did not substantially alter findings. Third, using education-specific or occupation-specific thresholds for poor cognitive performance in addition to thresholds based on age and sex had a small effect in improving predictive performance of cognitive impairment for dementia. Fourth, in general, cognitive impairment had a weaker association with mortality than with dementia. Finally, a model including only demographic variables (ie, age, sex, and education) was a substantially better predictor of dementia than all seven cognitive impairment definitions and thus adding these demographic measures to the prediction model substantially improved prediction accuracy.

Much of the research on cognitive impairment focuses on conversion rates to dementia. A 2009 meta-analysis reported the cumulative conversion rate to be 22·7% (95% CI 14·2–32·6) in community settings.^23^ In our study, with assessment of cognitive decline over 10 years and a subsequent 10-year follow-up, the cumulative conversion ranged from 9% to 18% for the seven definitions of cognitive impairment. Conversion to dementia might not be meaningful, because an annual conversion rate of 10% from MCI to dementia reported in a review^24^ seems implausible because it implies that nearly two thirds of the study population will have converted to dementia after 10 years. Furthermore, the review also reported considerable heterogeneity in conversion rates between studies, limiting the usefulness of MCI for identifying risk of dementia. Two recent studies used data from the Alzheimer's Disease Neuroimaging Initiative study, based only on people with a diagnosis of MCI, to examine conversion to dementia. One study found cognitive measures to be more robust predictors of conversion than cerebrospinal fluid biomarkers,^25^ and the second study used more than 750 predictors and found the best-performing model to include demographic measures, cognitive and functional markers, and morphometric MRI measures (83% sensitivity, 76% specificity, area under the curve [AUC] 0·87).^26^ As in our study, the role of demographic variables in prediction has also been noted in a recent study that examined conversion from normal cognition to MCI, showing that models based on demographic variables alone had high predictive accuracy, with an AUC of 0·68.^27^ Taken together, these findings highlight the importance of demographic variables in dementia prediction, which also have the advantage of being more widely available than cerebrospinal fluid or imaging biomarkers.

An important element in the conceptualisation of cognitive impairment is loss of cognition from a previous state, with recommendations that this should be assessed using serial measurement,^4^ preferably with a minimum of three datapoints.^7^ Cognitive testing at a single timepoint is less reliable and subject to random variation, particularly at older ages.^6^, ^7^ However, most studies do not use cognitive decline in the diagnosis of cognitive impairment, either because the study period is short or repeat data on cognitive function are not available. One exception is a study on 618 adults aged 70–90 years with data on cognitive decline over 2 years and a 6-year follow-up that found objective cognitive decline did not improve the predictive accuracy for progression to dementia (AUC 0·52–0·62 *vs* 0·59–0·72 for measures of poor cognitive performance).^28^ Another multicohort study on the progression from normal cognition to MCI instead reported better performance when including cognitive decline, in particular an improvement in specificity.^29^ Our results similarly show that including both poor cognitive performance and cognitive decline (definitions 3, 5, and 7) increases specificity, so these metrics should be used in contexts where it is important to avoid false-positive cases.

Education and occupation have been studied extensively in the onset of cognitive impairment and dementia, with a recent study showing education to delay the onset of cognitive impairment but not the subsequent progression to dementia.^30^ Likewise, our results suggest that using education-specific thresholds for poor cognitive performance has a marginal effect in terms of improving prediction of dementia. Taken together, our results support the position that cognitive impairment alone is a poor predictor of dementia.^23^, ^28^ While cognitive impairment definitions without demographic variables have high specificity, the low prevalence of dementia implies a large proportion of false-positive test results, resulting in low positive predictive value for cognitive impairment. A similar observation has been made for blood-based biomarkers for Alzheimer's disease that have high negative predictive value but low positive predictive value.^31^ Combining multiple predictors (such as biomarkers, imaging data, risk factors, and cognitive markers) to define cognitive impairment does improve prediction accuracy,^26^, ^32^, ^33^ but even in these studies predictive accuracy continues to stem primarily from demographic variables. Our results on cognitive impairment reflect findings on multivariable prediction models for dementia, whereby reviews of existing models have found their predictive accuracy to be poor.^34^

The primary strength of this study is the use of a large-scale, longitudinal study spanning over 20 years that allows assessment of the predictive value of cognitive impairment for dementia. Cognitive decline spanning 10 years could be used to determine cognitive impairment status, following recommendations in this domain.^4^, ^6^, ^7^ As such data are unlikely to be available in clinical practice, we also used measures of cognitive performance alone in definitions 1, 4, and 6. Other advantages were the use of an algorithm to guide diagnostic classification of cognitive impairment, which reduces random variability and biases due to variability in clinical judgment. Finally, the use of predictive statistics in addition to conversion rates and HRs to show strength of associations allows assessment of the utility of cognitive impairment.

Notwithstanding, when interpreting our results, the limitations of the study must also be taken into account. The cognitive battery did not include all possible cognitive domains, particularly measures of visuospatial memory or delayed recall, or multiple cognitive tests in each domain that might more accurately measure cognitive impairment. Ascertainment of dementia via linkage to electronic health records rather than clinical evaluation is likely to miss milder cases of dementia. However, this approach has the advantage of being able to include all participants in the analyses rather than just those with in-person ascertainment of dementia. In the UK, HES records on dementia have been shown to have high specificity but modest sensitivity (78%) due to milder cases of dementia being missed;^17^ to counter this, we additionally used data from community mental health services to improve the sensitivity of dementia diagnosis. Previous studies using these data on dementia have shown expected associations with risk factors,^11^ suggesting that the quality of the dementia data is unlikely to explain the findings of the present study. Furthermore, the presence of false negatives would affect all prediction models rather than only those using cognitive impairment. The analysis for dementia is based on smaller numbers, due to low incidence, and wider CIs for the HRs compared with the analysis for mortality; nonetheless, the number of events is large enough not to violate the guideline of 10–15 events per predictor. Low incidence did not allow examination of dementia-related mortality or dementia subtypes. Finally, Whitehall II study participants are likely to be healthier than the general population, although we have previously shown risk factor–outcome associations in the Whitehall II study to be similar to those in the general population.^35^

In conclusion, our study suggests that cognitive impairment might be necessary but not sufficient for later dementia. While addition of an objective measure of cognitive decline to the prediction model strengthened the HR of the association with dementia and improved specificity, the sensitivity in these models was lower, so that the predictive accuracy was similar to that in cognitive impairment definitions using only cognitive performance. It is worth noting, however, that cognitive decline had the best predictive accuracy for mortality. Further research is needed to determine risk factors or biomarkers that are useful in early identification of people at increased risk of dementia.

## Data sharing

Whitehall II data cannot be shared publicly because of constraints dictated by the study's ethics approval and institutional review board restrictions. The Whitehall II data are available for sharing within the scientific community. Researchers can apply for data access online.

## Declaration of interests

We declare no competing interests.
