# Supplementary material for: Comparison of the predictive accuracy of multiple definitions of cognitive impairment for incident dementia: a 20-year follow-up of the Whitehall II cohort study
Source: Lancet Healthy Longev. 2021 Jul;2(7):e407–16. doi: 10.1016/S2666-7568(21)00117-3 (PMC8245324; doi:10.1016/S2666-7568(21)00117-3)
Supplement: Supplementary appendix [file mmc1.pdf]

THE LANCET
Healthy Longevity

Supplementary appendix

This appendix formed part of the original submission and has been peer reviewed. We post it as supplied by the authors.

Supplement to: Machado-Fragua MD, Aline DugravotA, Dumurgier J, et al. Comparison of the predictive accuracy of multiple definitions of cognitive impairment for incident dementia: a 20-year follow-up of the Whitehall II cohort study. *Lancet Healthy Longev* 2021; published online xxx. https://doi.org/10.1016/S2666-7568(21)00117-3.

Comparison of the predictive accuracy of multiple definitions of cognitive impairment for incident dementia: A 20-year follow-up of the Whitehall II cohort study

Marcos D. Machado-Fragua, Aline Dugravot, Julien Dumurgier, Mika Kivimaki, Andrew Sommerlad, Benjamin Landré, Aurore Fayosse, Séverine Sabia, Archana Singh-Manoux

Stata syntax for the definition of poor cognitive performance (example: age and sex-specific threshold) 2

eTable 1. Kappa coefficients for agreement between seven cognitive impairment definitions 3

eTable 2. Assessment of the role of cognitive decline in classification of cognitive impairment: association with risk of dementia...4

eTable 3. Predictive performance of seven cognitive impairment definitions for dementia using inverse probability weighting to account for missing data 5

eTable 4. Predictive performance of seven cognitive impairment definitions for dementia using 1 SD threshold to define poor cognitive performance..6

eTable 5. Predictive performance of seven cognitive impairment definitions for dementia using 1.96 SD threshold to define poor cognitive performance SD 7

eTable 6. Predictive performance of seven cognitive impairment definitions for dementia in participants 65 years and older at assessment of CI 8

eTable 7. Role of participants who revert their cognitive status to normal between 1997-2009 in classification of cognitive impairment: association with risk of dementia 9

eTable 8. Predictive performance of different predictive models including cognitive impairment definitions and demographic variables (age, sex, and education) for dementia 10

SUPPLEMENTAL METHODS

Stata syntax for the definition of poor cognitive performance (example: age and sex-specific threshold)

All thresholds were defined on distributions within the Whitehall II study population.

```
/* I:  Poor cognitive performance- Low Z-score                    */
/**************************************************/
/* LOW Z-SCORE : CONSTRUCTION */

/* ph: specific prefix of each data collection wave

Domain : standardized score of each cognitive domain

Phase: name of each data collection wave */


program zscore
args ph domain phase
regress `ph'`domain' `ph'age_si sex
predict `ph'`domain'_r, r
gen `ph'`domain'_rmse=e(rmse)
gen `ph'`domain'_z=`ph'`domain'_r/`ph'`domain'_rmse
gen `ph'`domain'_zlow=0
replace `ph'`domain'_zlow=1 if `ph'`domain'_z<=-1.5
replace `ph'`domain'_zlow=. if `ph'`domain'_z==.
label var `ph'`domain'_zlow "low score for `domain' domain (`phase')"
end

zscore j memory s9
zscore j language s9
zscore j executive s9
zscore j ScCog s9
```

eTable 1. Kappa coefficients for agreement between seven cognitive impairment definitions

**Cognitive Impairment**[a]	Definition 1	Definition 2	Definition 3	Definition 4	Definition 5	Definition 6	Definition 7
Definition 1	-	0·13	0·54	0·79	0·46	0·69	0·41
Definition 2	-	-	0·30	0·14	0·31	0·15	0·32
Definition 3	-	-	-	0·46	0·86	0·42	0·78
Definition 4	-	-	-	-	0·55	0·72	0·44
Definition 5	-	-	-	-	-	0·45	0·83
Definition 6	-	-	-	-	-	-	0·56
Definition 7	-	-	-	-	-	-	-

[a] Cognitive Impairment defined as follows:

1. Age and sex threshold for poor cognitive performance.
2. Cognitive decline.
3. Age, sex threshold for poor cognitive performance AND cognitive decline.
4. Age, sex, education threshold for poor cognitive performance.
5. Age, sex, education threshold for poor cognitive performance AND cognitive decline.
6. Age, sex, occupation threshold for poor cognitive performance.
7. Age, sex, occupation threshold for poor cognitive performance AND cognitive decline.

eTable 2. Assessment of the role of cognitive decline in classification of cognitive impairment: association with risk of dementia

Cognitive Impairment (CI)[a]	N cases/total	Rate of dementia per 1000 person years	Follow-up years M(SD)	HR (95% Confidence Interval)	*p*-value[b]
**Age and sex threshold for poor cognitive performance**					
Non-CI	179/4866	3·7	10·1 (1·7)	1 (Ref.)	
Poor cognitive performance only	31/483	6·5	9·9 (2·0)	1·79 (1·22, 2·61)	
Poor cognitive performance & cognitive decline	60/338	19·8	9·0 (2·9)	5·67 (4·23, 7·60)	<·0001
**Age, sex, and education threshold for poor cognitive performance**					
Non-CI	175/4850	3·6	10·1 (1·7)	1 (Ref.)	
Poor cognitive performance only	32/490	6·6	9·9 (2·0)	1·85 (1·27, 2·70)	
Poor cognitive performance & cognitive decline	63/347	20·2	9·0 (2·9)	5·90 (4·42, 7·87)	<·0001
**Age, sex, and occupation threshold for poor cognitive performance**					
Non-CI	178/4861	3·6	10·1 (1·8)	1 (Ref.)	
Poor cognitive performance only	31/472	6·6	10·0 (1·9)	1·81 (1·24, 2·65)	
Poor cognitive performance & cognitive decline	61/354	19·0	9·0 (2·9)	5·44 (4·07, 7·28)	<·0001

HR: Hazard Ratio, M(SD)=Mean (Standard Deviation).

[a] Cognitive impairment categories are mutually exclusive.

[b] p-value for the difference between poor cognitive performance only and poor cognitive performance & definite cognitive decline.

eTable 3. Predictive performance of seven cognitive impairment definitions for dementia using inverse probability weighting to account for missing data

Cognitive Impairment (CI) definitions	N cases /total	Rate of dementia/ 1000 person-years	Follow-up years M(SD)	HR (95% Confidence Interval)	R^2 (95% Confidence Interval)	AIC	Δ AIC	Sensitivity %	Specificity %	C-Statistic (95% Confidence Interval)	*p*-value[a]
**1. Age and sex threshold for poor cognitive performance**											
Non-CI	179/4866	4·6	10·1 (1·7)	1 (Ref.)	0·219	5959·5	Ref.	32·9	86·6	0·621	Ref.
CI	91/821	14·6	9·5 (2·4)	3·23 (2·47, 4·23)	(0·119, 0·338)					(0·588, 0·653)	
**2. Cognitive decline**											
Non-CI	136/4202	4·4	10·1 (1·7)	1 (Ref.)	0·180	5978·6	19·1	48·4	75·1	0·618	·88
CI	134/1485	11·7	9·6 (2·3)	2·70 (2·09, 3·48)	(0·099, 0·280)					(0·586, 0·650)	
**3. Age, sex threshold for poor cognitive performance AND cognitive decline**											
Non-CI	210/5349	5·1	10·1 (1·8)	1 (Ref.)	0·258	5939·5	- 20·0	21·8	94·9	0·597	·07
CI	60/338	23·9	9·0 (2·9)	4·94 (3·64, 6·70)	(0·140, 0·379)					(0·569, 0·626)	
**4. Age, sex, education threshold for poor cognitive performance**											
Non-CI	175/4850	4·5	10·1 (1·7)	1 (Ref.)	0·243	5947·3	- 12·2	34·3	86·4	0·625	·63
CI	95/837	15·1	9·5 (2·5)	3·43 (2·63, 4·47)	(0·143, 0·364)					(0·593, 0·657)	
**5. Age, sex, education threshold for poor cognitive performance AND cognitive decline**											
Non-CI	207/5340	5·1	10·1 (1·8)	1 (Ref.)	0·264	5936·5	- 23·0	22·4	94·8	0·598	·10
CI	63/347	24·1	9·0 (2·9)	4·99 (3·69, 6·74)	(0·150,0·390)					(0·569, 0·626)	
**6. Age, sex, occupation threshold for poor cognitive performance**											
Non-CI	178/4861	4·8	10·1 (1·8)	1 (Ref.)	0·206	5965·6	6·1	32·5	86·5	0·613	·52
CI	92/826	14·9	9·6 (2·4)	3·20 (2·45, 4·19)	(0·110, 0·315)					(0·582, 0·645)	
**7. Age, sex, occupation threshold for poor cognitive performance AND cognitive decline**											
Non-CI	209/5333	5·2	10·1 (1·8)	1 (Ref.)	0·246	5945·7	- 13·8	21·6	94·6	0·592	·06
CI	61/354	23·8	9·0 (2·9)	4·83 (3·56, 6·55)	(0·135, 0·373)					(0·564, 0·620)	

HR: Hazard Ratio, CI: Cognitive impairment, R^2=Royston's R^2, AIC: Akaike information criterion, C-Statistic=Harrell's C-index, M(SD)=Mean (Standard Deviation).

[a]*p* values for difference in C-statistic.

eTable 4. Predictive performance of seven cognitive impairment definitions for dementia using 1 SD threshold to define poor cognitive performance

Cognitive Impairment (CI) definitions	N cases /total	Rate of dementia/ 1000 person-years	Follow-up years M(SD)	HR (95% Confidence Interval)	R^2 (95% Confidence Interval)	AIC	Δ AIC	Sensitivity %	Specificity %	C-Statistic (95% Confidence Interval)	*p*-value[a]
**1. Age and sex threshold for poor cognitive performance**											
Non-CI	126/3954	3·2	10·1 (1·7)	1 (Ref.)	0·146	4532·1	Ref.	53·0	70·8	0·624	Ref.
CI	144/1733	8·5	9·7 (2·2)	2·74 (2·16, 3·48)	(0·087, 0·220)					(0·594, 0·654)	
**2. Cognitive decline**											
Non-CI	136/4202	3·2	10·1 (1·7)	1 (Ref.)	0·169	4521·4	- 10·7	48·4	75·1	0·631	·73
CI	134/1485	9·4	9·6 (2·3)	3·01 (2·37, 3·82)	(0·102, 0·248)					(0·601, 0·660)	
**3. Age, sex threshold for poor cognitive performance AND cognitive decline**											
Non-CI	186/5065	3·6	10·1 (1·7)	1 (Ref.)	0·201	4505·7	- 26·4	30·5	90·1	0·612	·34
CI	84/622	14·6	9·3 (2·6)	4·14 (3·20, 5·36)	(0·123, 0·286)					(0·584, 0·640)	
**4. Age, sex, education threshold for poor cognitive performance**											
Non-CI	127/3920	3·2	10·1 (1·7)	1 (Ref.)	0·135	4537·2	5·1	52·0	70·1	0·621	·72
CI	143/1767	8·3	9·7 (2·2)	2·63 (2·07, 3·34)	(0·076, 0·209)					(0·591, 0·651)	
**5. Age, sex, education threshold for poor cognitive performance AND cognitive decline**											
Non-CI	186/5031	3·7	10·1 (1·7)	1 (Ref.)	0·185	4513·4	- 18·7	30·2	89·5	0·609	·29
CI	84/656	13·8	9·3 (2·6)	3·87 (2·99, 5·01)	(0·108, 0·272)					(0·581, 0·637)	
**6. Age, sex, occupation threshold for poor cognitive performance**											
Non-CI	117/3829	3·0	10·1 (1·7)	1 (Ref.)	0·157	4527·2	- 4·9	56·6	68·6	0·629	·60
CI	153/1858	8·4	9·8 (2·1)	2·83 (2·23, 3·60)	(0·093, 0·234)					(0·600, 0·659)	
**7. Age, sex, occupation threshold for poor cognitive performance AND cognitive decline**											
Non-CI	179/5004	3·5	10·1 (1·7)	1 (Ref.)	0·214	4499·1	- 33·0	33·2	89·2	0·619	·76
CI	91/683	14·3	9·3 (2·6)	4·17 (3·24, 5·37)	(0·131, 0·302)					(0·591, 0·648)	

HR: Hazard Ratio, CI: Cognitive impairment, R^2=Royston's R^2, AIC: Akaike information criterion, C-Statistic=Harrell's C-index, M(SD)=Mean (Standard Deviation).
[a]*p* values for difference in C-statistic.

eTable 5. Predictive performance of seven cognitive impairment definitions for dementia using 1.96 SD threshold to define poor cognitive performance SD

Cognitive Impairment (CI) definitions	N cases /total	Rate of dementia/ 1000 person-years	Follow-up years M(SD)	HR (95% Confidence Interval)	R^2 (95% Confidence Interval)	AIC	Δ AIC	Sensitivity %	Specificity %	C-Statistic (95% Confidence Interval)	*p*-value[a]
**1. Age and sex threshold for poor cognitive performance**											
Non-CI	213/5328	4·0	10·0 (1·8)	1 (Ref.)	0·159	4526·2	Ref.	20·7	94·5	0·581	Ref.
CI	57/359	17·1	9·3 (2·7)	4·41 (3·29, 5·91)	(0·086, 0·247)					(0·556, 0·606)	
**2. Cognitive decline**											
Non-CI	136/4202	3·2	10·1 (1·7)	1 (Ref.)	0·169	4521·4	- 4·8	48·4	75·1	0·631	·006
CI	134/1485	9·4	9·6 (2·3)	3·01 (2·37, 3·82)	(0·102, 0·248)					(0·601, 0·660)	
**3. Age, sex threshold for poor cognitive performance AND cognitive decline**											
Non-CI	230/5520	4·2	10·0 (1·8)	1 (Ref.)	0·180	4515·9	- 10·3	14·7	97·7	0·565	·04
CI	40/167	27·8	8·6 (3·1)	7·09 (5·07, 9·93)	(0·094, 0·284)					(0·544, 0·588)	
**4. Age, sex, education threshold for poor cognitive performance**											
Non-CI	214/5304	4·0	10·0 (1·8)	1 (Ref.)	0·139	4535·5	9·3	20·3	94·0	0·577	·47
CI	56/383	15·6	9·4 (2·7)	3·98 (2·97, 5·34)	(0·072, 0·222)					(0·552, 0·602)	
**5. Age, sex, education threshold for poor cognitive performance AND cognitive decline**											
Non-CI	232/5511	4·2	10·0 (1·8)	1 (Ref.)	0·152	4529·2	3·0	13·9	97·5	0·561	·02
CI	38/176	24·5	8·8 (3·0)	6·11 (4·34, 8·61)	(0·082, 0·243)					(0·539, 0·582)	
**6. Age, sex, occupation threshold for poor cognitive performance**											
Non-CI	216/5333	4·0	10·0 (1·8)	1 (Ref.)	0·143	4533·4	7·2	18·9	94·5	0·576	·54
CI	54/354	16·5	9·3 (2·7)	4·20 (3·12, 5·66)	(0·074, 0·228)					(0.551, 0·600)	
**7. Age, sex, occupation threshold for poor cognitive performance AND cognitive decline**											
Non-CI	227/5504	4·1	10·0 (1·8)	1 (Ref.)	0·189	4511·3	- 14·9	15·3	97·4	0·569	·21
CI	43/183	27·0	8·7 (3·0)	6·97 (5·03, 9·66)	(0·106, 0·284)					(0·547, 0·591)	

HR: Hazard Ratio, CI: Cognitive impairment, R^2=Royston’s R^2, AIC: Akaike information criterion, C-Statistic=Harrell’s C-index, M(SD)=Mean (Standard Deviation).
[a]*p* values for difference in C-statistic.

eTable 6. Predictive performance of seven cognitive impairment definitions for dementia in participants 65 years and older at assessment of CI

Cognitive Impairment (CI) definitions	N cases /total	Rate of dementia/ 1000 person-years	Follow-up years M(SD)	HR (95% Confidence Interval)	R^2 (95% Confidence Interval)	AIC	Δ AIC	Sensitivity %	Specificity %	C-Statistic (95% Confidence Interval)	*p*-value[a]
**1. Age and sex threshold for poor cognitive performance**											
Non-CI	157/2424	6·6	9·8 (2·1)	1 (Ref.)	0·145 (0·074, 0·234)	3551·0	Ref.	31·7	87·2	0·605 (0·574, 0·635)	Ref.
CI	75/413	20·3	9·0 (2·8)	3·18 (2·42, 4·19)							
**2. Cognitive decline**											
Non-CI	112/1978	5·7	9·9 (2·0)	1 (Ref.)	0·146 (0·076, 0·231)	3550·8	- 0·2	50·5	71·8	0·626 (0·594, 0·658)	·28
CI	120/859	15·2	9·2 (2·6)	2·75 (2·12, 3·56)							
**3. Age, sex threshold for poor cognitive performance AND cognitive decline**											
Non-CI	180/2637	7·0	9·8 (2·1)	1 (Ref.)	0·179 (0·092, 0·276)	3537·3	- 13·7	21·8	94·4	0·591 (0·563, 0·618)	·15
CI	52/200	30·9	8·4 (3·1)	4·71 (3·46, 6·41)							
**4. Age, sex, education threshold for poor cognitive performance**											
Non-CI	152/2404	6·5	9·8 (2·1)	1 (Ref.)	0·159 (0·088, 0·251)	3545·5	- 5·5	33·6	86·6	0·609 (0·578, 0·640)	·64
CI	80/433	20·5	9·0 (2·9)	3·28 (2·50, 4·30)							
**5. Age, sex, education threshold for poor cognitive performance AND cognitive decline**											
Non-CI	177/2630	6·9	9·8 (2·1)	1 (Ref.)	0·192 (0·106, 0·294)	3531·5	- 19·5	22·5	94·2	0·595 (0·566, 0·623)	·40
CI	55/207	31·5	8·4 (3·1)	4·84 (3·58, 6·55)							
**6. Age, sex, occupation threshold for poor cognitive performance**											
Non-CI	155/2420	6·5	9·8 (2·1)	1 (Ref.)	0·151	3548·7	- 2.3	31·6	87·0	0·607	·85
CI	77/417	20·5	9·0 (2·8)	3·22 (2·45, 4·24)	(0·079, 0·239)					(0·576, 0·638)	
**7. Age, sex, occupation threshold for poor cognitive performance AND cognitive decline**											
Non-CI	177/2625	6·9	9·8 (2·1)	1 (Ref.)	0·186	3534·1	- 16·9	22·5	94·0	0·594	·39
CI	55/212	30·7	8·5 (3·1)	4·69 (3·47, 6·35)	(0·104, 0·287)					(0·566, 0·622)	

HR: Hazard Ratio, CI: Cognitive impairment, R^2=Royston's R^2, AIC: Akaike information criterion, C-Statistic=Harrell's C-index, M(SD)=Mean (Standard Deviation).
[a]*p* values for difference in C-statistic**.**

eTable 7. Role of participants who revert their cognitive status to normal between 1997-2009 in classification of cognitive impairment: association with risk of dementia

Cognitive Impairment (CI)[a]	N cases/total	Rate of dementia per 1000 person years	Follow-up years M(SD)	HR (95% Confidence Interval)
**Age and sex threshold for poor cognitive performance**				
Non-CI	161/4399	3·6	10·1 (1·7)	1 (Ref.)
Non-CI (previous poor cognition)	18/467	3·8	10·0 (1·8)	1·06 (0·65, 1·73)
CI	91/821	11·6	9·5 (2·4)	3·27 (2·53, 4·24)
**Age, sex, and education threshold for poor cognitive performance**				
Non-CI	157/4345	3·6	10·1 (1·7)	1 (Ref.)
Non-CI (previous poor cognition)	18/505	3·6	10·0 (1·8)	1·00 (0·62, 1·63)
CI	95/837	11·9	9·5 (2·5)	3·40 (2·63, 4·38)
**Age, sex, and occupation threshold for poor cognitive performance**				
Non-CI	157/4066	3·7	10·1 (1·7)	1 (Ref.)
Non-CI (previous poor cognition)	21/638	3·3	10·0 (1·8)	0·89 (0·57, 1·41)
CI	92/826	11·6	9·6 (2·4)	3·20 (2·47, 4·14)

HR: Hazard Ratio, M(SD)=Mean (Standard Deviation).
[a] Cognitive impairment categories are mutually exclusive.

eTable 8. Predictive performance of different predictive models including cognitive impairment definitions and demographic variables (age, sex, and education) for dementia

	N cases /total	Rate of dementia/ 1000 person-years	Follow-up years M(SD)	HR (95% Confidence Interval)	R^2 (95% Confidence Interval)	AIC	Δ AIC	Sensitivity %	Specificity %	C-Statistic (95% Confidence Interval)	*p*-value[a]	*p*-value[b]
**PREDICTIVE MODELS**												
**Model 1: Age and sex threshold for poor cognitive performance + demographic variables (age, sex, and education)**												
Non-CI	179/4866	3·7	10·1 (1·7)	1 (Ref.)	0·643	4235·0	Ref.	77·2	69·8	0·807	Ref.	<·0001
CI	91/821	11·6	9·5 (2·4)	3·57 (2·76, 4·61)	(0·565, 0·728)					(0·783, 0·831)		
**Model 2: Cognitive decline + demographic variables (age, sex, and education)**												
Non-CI	136/4202	3·2	10·1 (1·7)	1 (Ref.)	0·606	4266·5	31·5	79·5	68·2	0·802	·47	<·0001
CI	134/1485	9·4	9·6 (2·3)	2·38 (1·87, 3·03)	(0·526, 0·688)					(0·777, 0·827)		
**Model 3: Age, sex threshold for poor cognitive performance AND cognitive decline + demographic variables (age, sex, and education)**												
Non-CI	210/5349	3·9	10·1 (1·8)	1 (Ref.)	0·644	4234·4	- 0·6	76·1	71·7	0·803	·40	·001
CI	60/338	19·8	9·0 (2·9)	4·67 (3·49, 6·24)	(0·567, 0·727)					(0·778, 0·828)		
**Model 4: Age, sex, education threshold for poor cognitive performance + demographic variables (age, sex, and education)**												
Non-CI	175/4850	3·6	10·1 (1·7)	1 (Ref.)	0·647	4231·9	- 3·1	79·8	68·2	0·807	·89	<·0001
CI	95/837	11·9	9·5 (2·5)	3·56 (2·77, 4·58)	(0·565, 0·728)					(0·782, 0·831)		
**Model 5: Age, sex, education threshold for poor cognitive performance AND cognitive decline + demographic variables (age, sex, and education)**												
Non-CI	207/5340	3·9	10·1 (1·8)	1 (Ref.)	0·651	4228·1	- 6·9	78·4	69·4	0·804	·52	<·0001
CI	63/347	20·2	9·0 (2·9)	4·79 (3·61, 6·37)	(0·573, 0·732)					(0·779, 0·829)		
**Model 6: Age, sex, occupation threshold for poor cognitive performance + demographic variables (age, sex, and education)**												
Non-CI	178/4861	3·6	10·1 (1·8)	1 (Ref.)	0·640	4237·8	2·8	79·4	68·5	0·807	·93	<·0001
CI	92/826	11·6	9·6 (2·4)	3·42 (2·65, 4·40)	(0·562, 0·720)					(0·782, 0·831)		
**Model 7: Age, sex, occupation threshold for poor cognitive performance AND cognitive decline + demographic variables (age, sex, and education)**												
Non-CI	209/5333	3·9	10·1 (1·8)	1 (Ref.)	0·641	4236·9	1·9	76·6	71·1	0·801	·28	<·0001
CI	61/354	19·0	9·0 (2·9)	4·45 (3·34, 5·93)	(0·563, 0·727)					(0·775, 0·827)		
**DEMOGRAPHIC MODEL**												
**Age, sex, & education**	270/5687	NA	NA	NA	0·543 (0·465, 0·626)	4312·6	77·6	74·2	72·2	0·783 (0·758, 0·809)	<·0001	Ref.

HR: Hazard Ratio, CI: Cognitive impairment, R^2=Royston's R^2, AIC: Akaike information criterion, C-Statistic=Harrell's C-index, M(SD)=Mean (Standard Deviation).

[a]*p* values for difference in C-statistic using definition 1 as reference.

[b]*p* values for difference in C-statistic using demographic model as reference.
